# Supplementary material for: SyNDI: synchronous network data integration framework
Source: BMC Bioinformatics. 2018 Nov 6;19:403. doi: 10.1186/s12859-018-2426-5 (PMC6219086; doi:10.1186/s12859-018-2426-5)
Supplement: Supplementary file 5 — Files of the example presented in Additional file 4. This additional file is a zip package that contains all files used in Additional file 4. Detailed descriptions of each of these files are presented in a README file included in this zip package. (GZ 30411 kb) [file 12859_2018_2426_MOESM5_ESM.gz › SyNDITools/Meme2Fimo/meme_4.9.0_4.tar.gz!/meme_4.9.0/website/cgi-bin/job_status.tmpl]

<!-- TMPL\_VAR NAME=program --> Job<!-- TMPL\_IF NAME=status --> - <!-- TMPL\_VAR NAME=status --><!-- /TMPL\_IF -->


## Job -

This page will be updated every  seconds. You may bookmark this page and return to it later.

### Results


### Status Messages
